# Supplementary material for: USP21 prevents the generation of T-helper-1-like Treg cells
Source: Nat Commun. 2016 Nov 18;7:13559. doi: 10.1038/ncomms13559 (PMC5120220; doi:10.1038/ncomms13559)
Supplement: Supplementary Information — Supplementary Figures 1-8. [file ncomms13559-s1.pdf]

Supplementary Figure 1

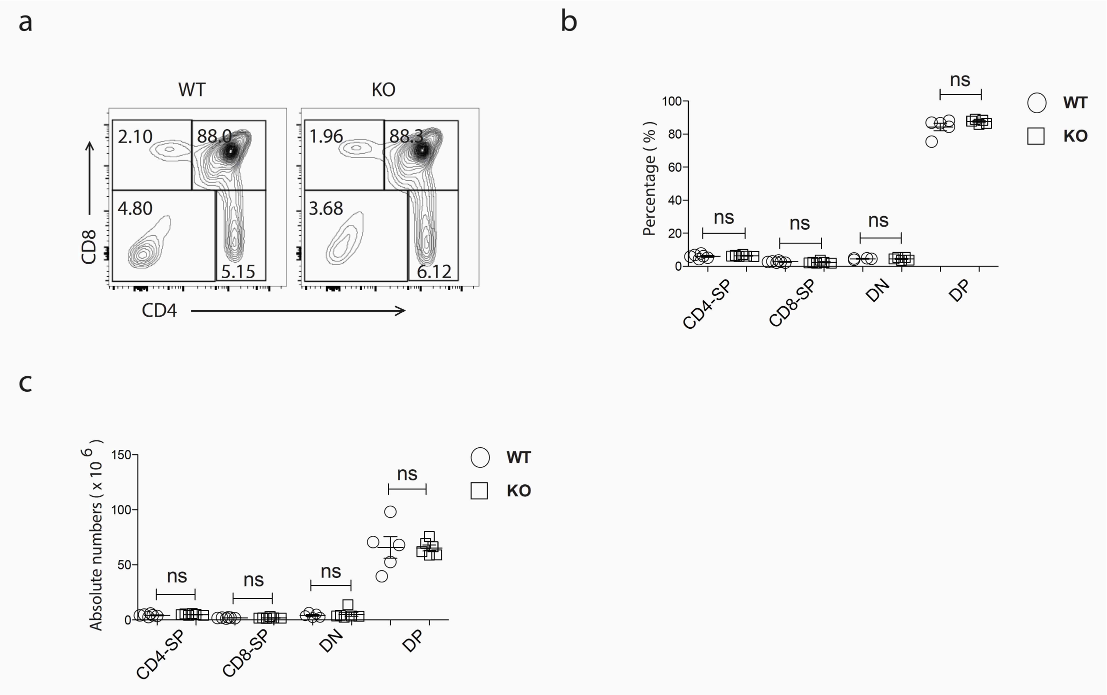

Supplementary Figure 1. **Unaltered thymic development of T cells in USP21-ΔTreg mice**. a, Representative figure shown the distribution of $CD4^{+}CD8^{-}$ (CD4-SP) thymocytes, $CD4^{-}CD8^{+}$ (CD8-SP) thymocytes, $CD4^{-}CD8^{-}$ (DN) thymocytes and $CD4^{+}CD8^{+}$ (DP) thymocytes in *Foxp3*^{Cre} (WT, n=5) and *Usp21*$^{fl/fl}$*Foxp3*^{Cre} (KO, n=6) mice. b, Percentage of CD4-SP, CD8-SP, DN and DP thymocytes in WT (n=5) and KO (n=6) mice as shown in a. c, Absolute numbers of CD4-SP, CD8-SP, DN and DP thymocytes in WT (n=5) and KO (n=6) mice as shown in a. All data represent means ± s.d. ns, not significant, as determined by Student's t-test.

Supplementary Figure 2

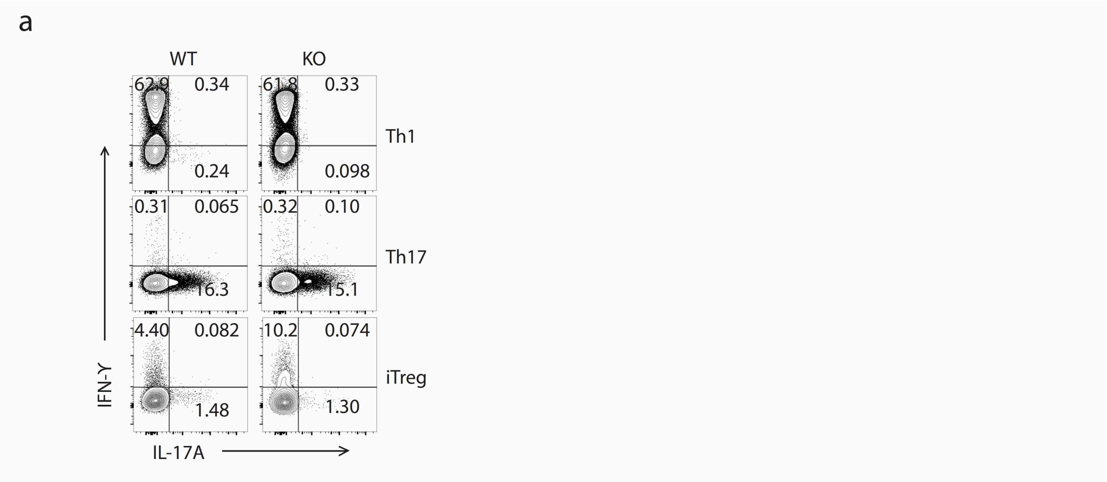

Supplementary Figure 2. **USP21-deficient iTreg cells displayed a Th1-like phenotype**. a, $CD4^{+}CD25^{-}YFP^{-}CD62L^{hi}$ naive T were sorted from WT and KO mice and further polarized into Th1, Th17 and iTreg cells. The expression of IFN-γ and IL-17 was further analyzed in Th1, Th17 and iTreg cells.

Supplementary Figure 3

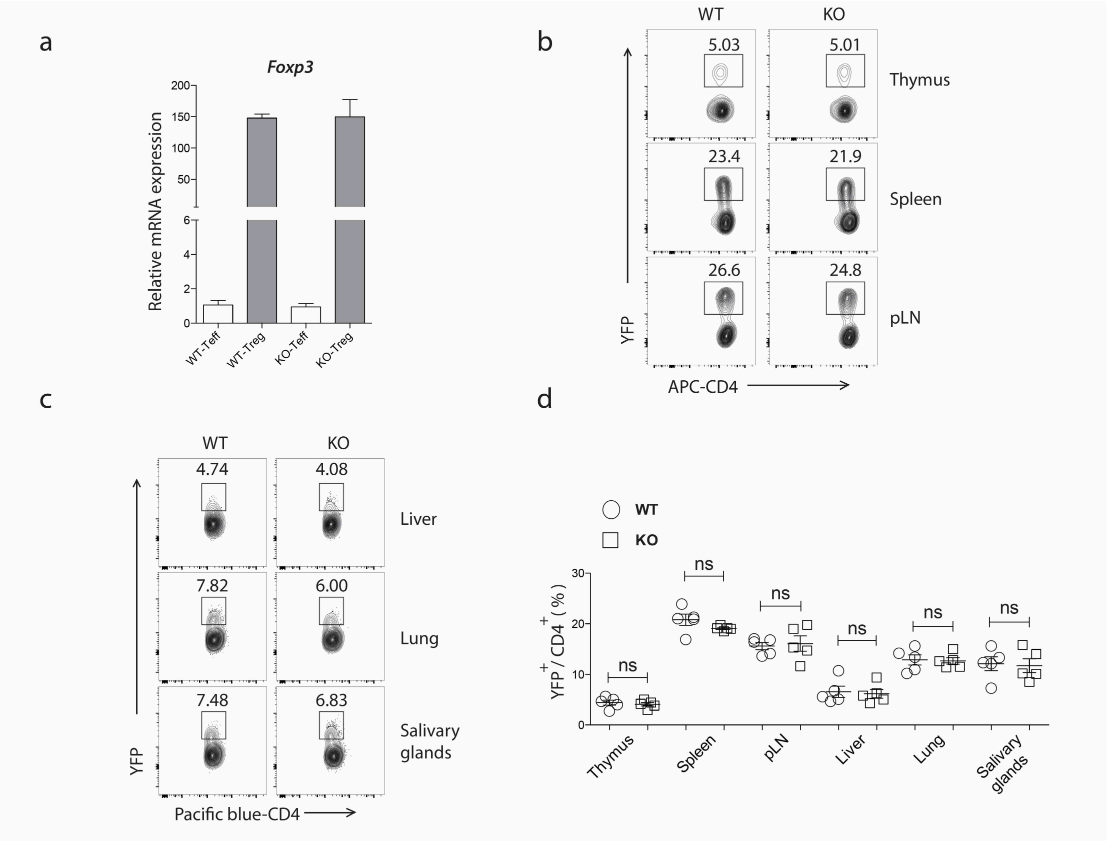

Supplementary Figure 3. **Stable expression of Foxp3 mRNA and YFP in USP21-ΔTreg cells**. a, $CD4^{+}CD25^{-}YFP^{-}$ effector T (Teff) cells and $CD4^{+}CD25^{hi}YFP^{+}$ Treg cells were sorted from WT and KO mice. mRNA expression of *Foxp3* in each population was assessed by qRT-PCR. (n=3 for each group). b, Representative figure shown the expression of YFP in $CD4^{+}$ T cells from the thymus, spleen and pLNs of WT (n=5) and KO (n=5) mice. c, Representative figure shown the expression of YFP in $CD4^{+}$ T cells from the liver, lung and salivary glands of WT (n=5) and KO (n=5) mice. d, Percentage of YFP^{+} Treg cells from the thymus, spleen, pLNs, liver, lung and salivary glands of WT and KO littermates, as indicated in b and c. All data represent means ± s.d. ns, not significant, as determined by Student’s t-test.

Supplementary Figure 4

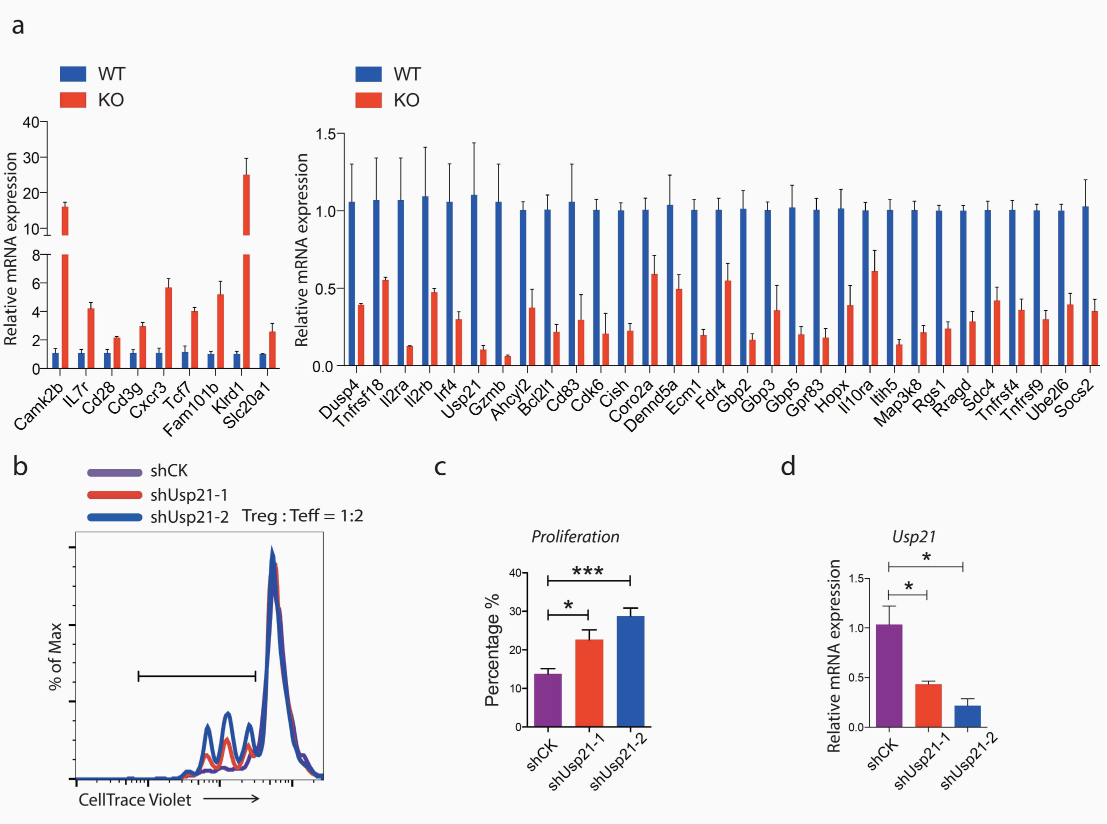

Supplementary Figure 4. USP21 maintains the expression of Treg signature genes and suppressive activity of Treg cells. a, Validation of the expression of representative genes significantly upregulated or downregulated in USP21-ΔTreg cells. (n=3 for each group). Data represent means ± s.d. b, $CD4^{+}CD25^{hi}YFP^{+}$ Treg cells were transduced with virus containing shRNA sequences targeting CK (control) or *Usp21*. Cells were cultured with anti-CD3/CD28 dynal beads and selected with puromycin for 2 days. Selected Treg cells were tested using a suppression assay. c, Percentage of proliferated responder T cells was assessed as shown in b. (n=6 for each group). Data represent means ± s.d. d, Knockdown efficiency of *Usp21* in Treg cells as indicated in b. (n=3 for each group). Data represent means ± s.d. *P≤0.05, ***P≤0.001, as determined by Student's t-test. ns, not significant.

Supplementary Figure 5

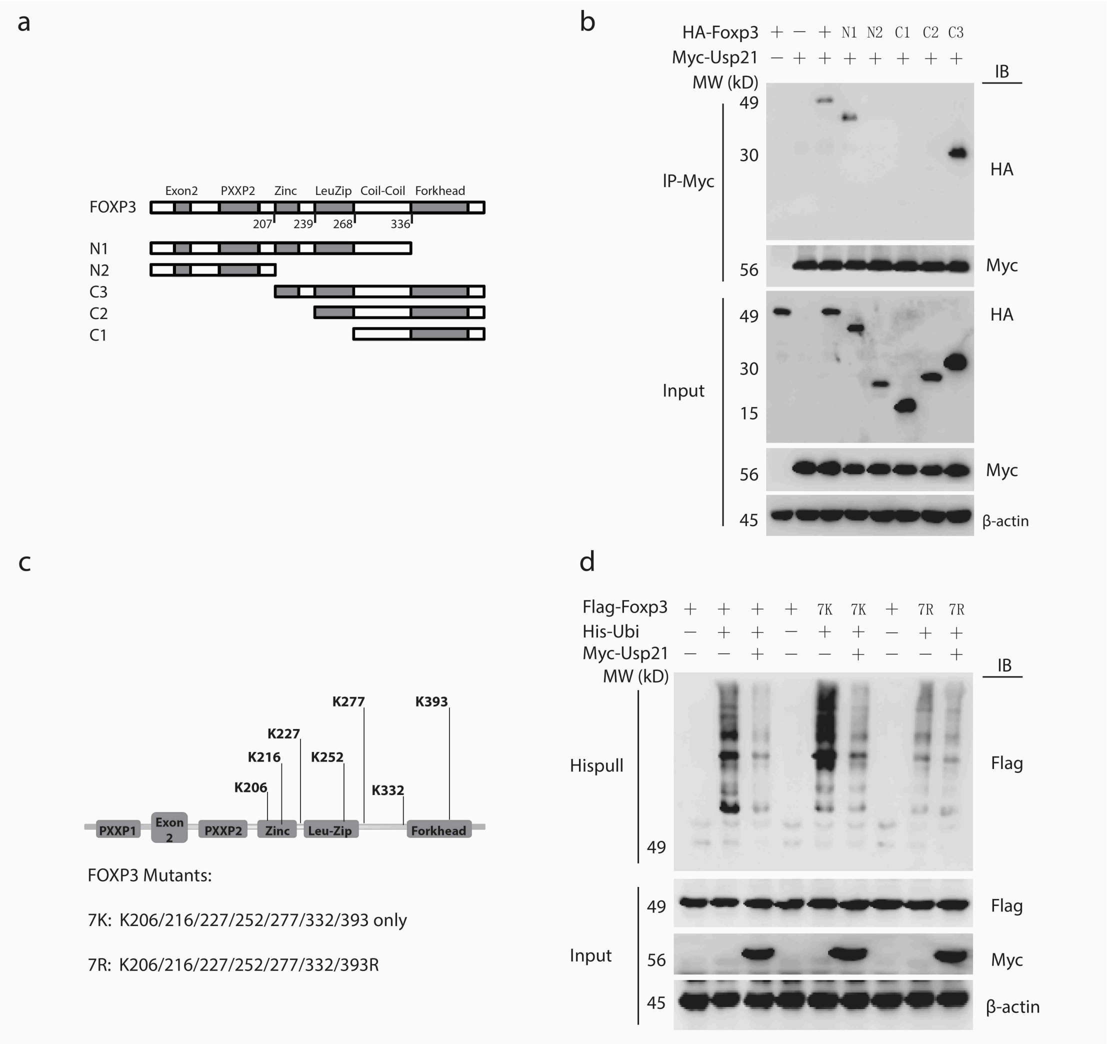

Supplementary Figure 5. USP21 interacts with the zinc finger subdomain of FOXP3 and deubiquitinates FOXP3 at seven lysine residues. a and b, Various truncated HA-Foxp3 constructs were generated as shown, and were cotransfected with or without Myc-Usp21 into 293T cells. Cell lysates were immunoprecipitated using anti-Myc antibody and FOXP3 levels were detected by Western blotting. c, Image indicating the domain structure of FOXP3 and the lysine residues deubiquitinated by USP21. FOXP3 7K and 7R mutants were described as indicated. d, His-ubiquitin pull-down assay of Flag-FOXP3 and FOXP3 7K or 7R mutants.

Supplementary Figure 6

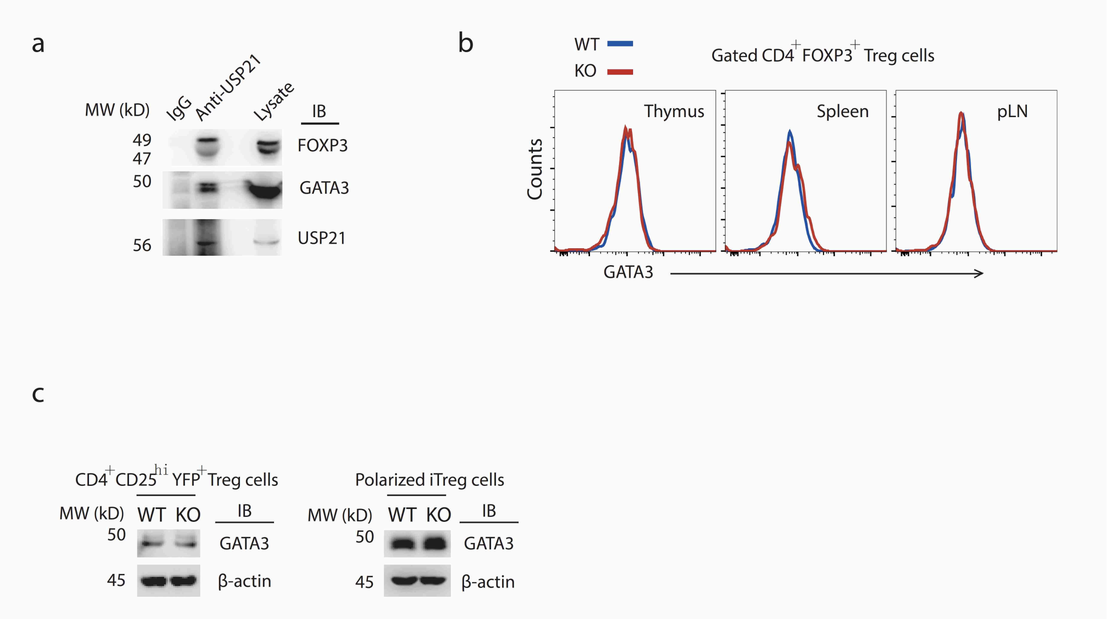

Supplementary Figure 6. USP21 is dispensable for GATA3 expression in murine Tregs. a, Human CD4^{+}CD25^{hi}CD127^{lo} expanding Treg cells were cultured with α-CD3 and α-CD28 antibodies in the presence of IL-2. Cell lysates were immunoprecipitated with anti-USP21 antibody; GATA3 or FOXP3 levels were detected by Western blotting. b, Expression of GATA3 in CD4^{+}FOXP3^{+} Treg cells from the thymus, spleen and pLNs of WT and KO mice. c, Expression of GATA3 protein in CD4^{+}CD25^{hi}YFP^{+} Treg cells or polarized iTreg cells from WT and KO mice.

Supplementary Figure 7

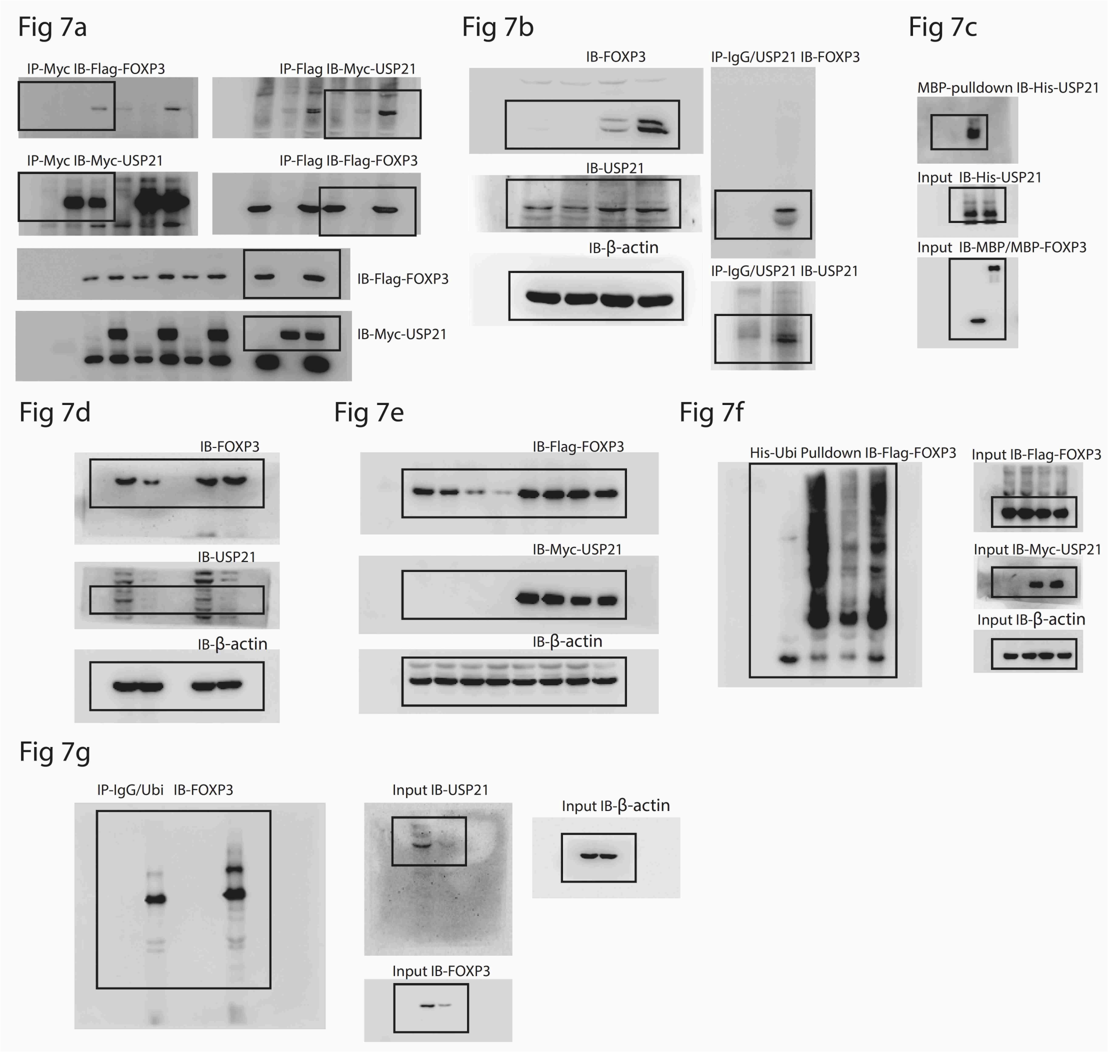

Supplementary Figure 7. The uncropped scans of western blots from Figure 7.

Supplementary Figure 8

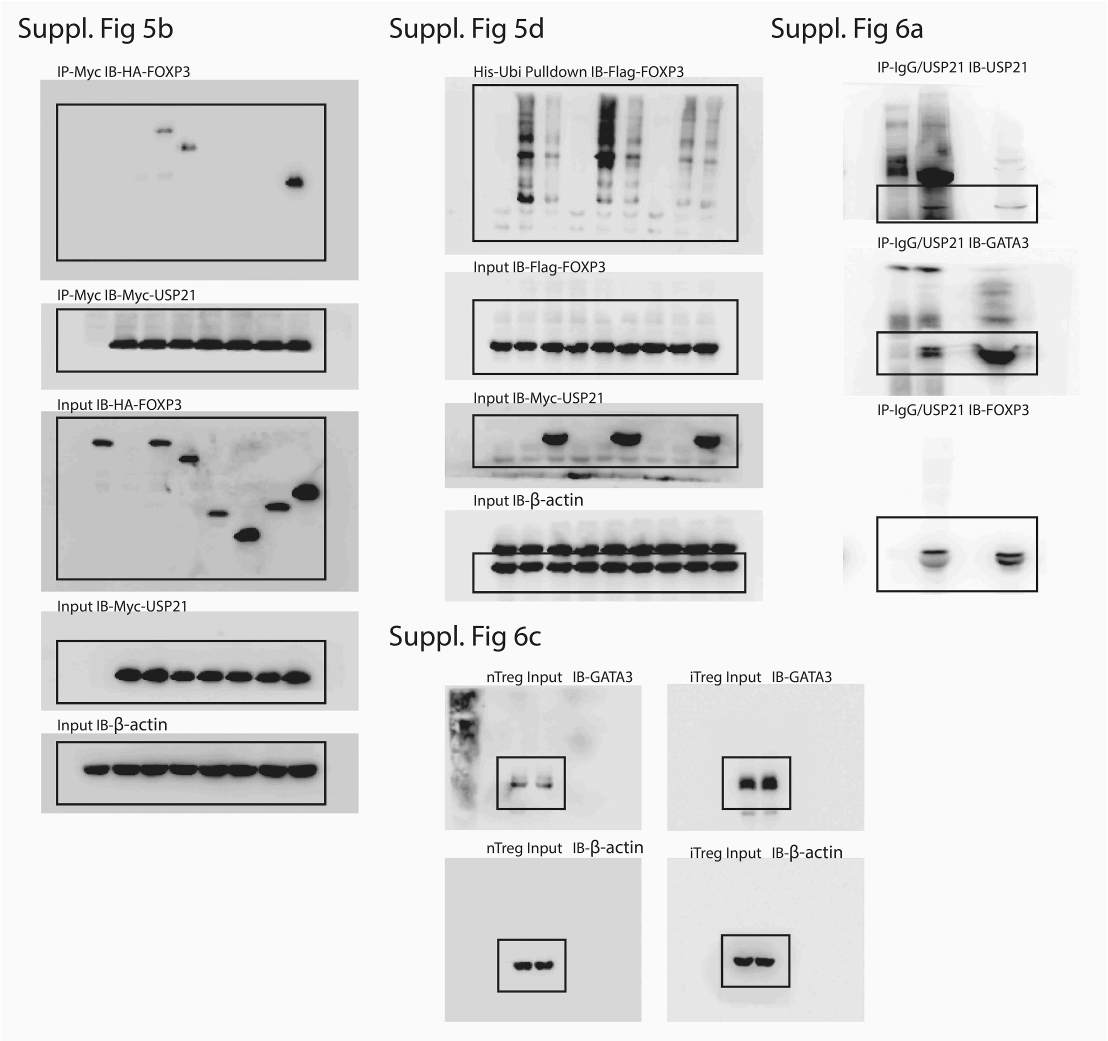

Supplementary Figure 8. The uncropped scans of western blots from Supplementary Figure 5 and 6.
